# Electrophysiological correlates of basic semantic composition in people with aphasia

**DOI:** 10.1016/j.nicl.2023.103516

**Published:** 2023-09-23

**Authors:** Astrid Graessner, Caroline Duchow, Emiliano Zaccarella, Angela D. Friederici, Hellmuth Obrig, Gesa Hartwigsen

**Affiliations:** a Wilhelm Wundt Institute for Psychology, Leipzig University, Germany; b Lise-Meitner Research Group Cognition and Plasticity, Max Planck Institute for Human Cognitive and Brain Sciences, Leipzig, Germany; c Department of Neuropsychology, Max Planck Institute for Human Cognitive and Brain Sciences, Leipzig, Germany; d Clinic for Cognitive Neurology, University Hospital Leipzig, Germany; e Department of Neurology, Max Planck Institute for Human Cognitive and Brain Sciences, Leipzig, Germany

**Keywords:** Meaning composition, ERP, N400, Aphasia

## Abstract

The neuroanatomical correlates of basic semantic composition have been investigated in previous neuroimaging and lesion studies, but research on the electrophysiology of the involved processes is scarce. A large literature on sentence-level event-related potentials (ERPs) during semantic processing has identified at least two relevant components – the N400 and the P600. Other studies demonstrated that these components are reduced and/or delayed in people with aphasia (PWA). However, it remains to be shown if these findings generalize beyond the sentence level. Specifically, it is an open question if an alteration in ERP responses in PWA can also be observed during basic semantic composition, providing a potential future diagnostic tool.

The present study aimed to elucidate the electrophysiological dynamics of basic semantic composition in a group of post-stroke PWA. We included 20 PWA and 20 age-matched controls (mean age 58 years) and measured ERP responses while they performed a plausibility judgment task on two-word phrases that were either meaningful (“anxious horse”), anomalous (“anxious wood”) or had the noun replaced by a pseudoword (“anxious gufel”).

The N400 effect for anomalous versus meaningful phrases was similar in both groups. In contrast, unlike the control group, PWA did not show an N400 effect between pseudoword and meaningful phrases. Moreover, both groups exhibited a parietal P600 effect towards pseudoword phrases, while PWA showed an additional P600 over frontal electrodes. Finally, PWA showed an inverse correlation between the magnitude of the N400 and P600 effects: PWA exhibiting no or even reversed N400 effects towards anomalous and pseudoword phrases showed a stronger P600 effect. These results may reflect a compensatory mechanism which allows PWA to arrive at the correct interpretation of the phrase. When compositional processing capacities are impaired in the early N400 time-window, PWA may make use of a more elaborate re-analysis process reflected in the P600.

## Highlights

• People with aphasia show no N400 effect towards minimal pseudoword phrases.
• People with aphasia may show a compensatory late positivity.
• No difference between people with aphasia and healthy controls for minimal anomalous phrases.

## 1 Introduction

Language comprehension relies on the rapid composition of single words’ meanings into more complex semantic representations. The demands of semantic composition depend on the plausibility and likelihood of concepts to co-occur. Neurolinguistic research of the past decades has made significant progress in understanding the temporal and spatial brain bases underlying semantic composition (Bemis and Pylkkänen, 2013, Brennan and Pylkkänen, 2012, Fló et al., 2020, Humphries et al., 2006, Lau et al., 2008, Matchin et al., 2017, Pallier et al., 2011, Vandenberghe et al., 2002, Vigneau et al., 2006). Moreover, it has been recognized that deficits in language comprehension in people with aphasia (PWA) do not only arise at the lexical or syntactic level but may also impair the integration of meaning across individual words. In this vein, research on the electrophysiological correlates of aphasic language processing has shown that the event-related-potential (ERP) component N400, related to the lexico-semantic processing of an utterance, is diminished and/or delayed as compared to age-matched controls (Chang et al., 2016, Kawohl et al., 2010, Khachatryan et al., 2017, Sheppard et al., 2017, Swaab et al., 1997), for a review see Meechan et al. (2021). Notably, the N400 amplitude in response to a highly implausible last word of a sentence has been shown to be decreased with increasing aphasia severity (Chang et al., 2016, Kawohl et al., 2010, Swaab et al., 1997, Hagoort et al., 1996). (For a more detailed discussion see below).

Since its original discovery (Kutas and Hillyard, 1980), the N400 has been extensively studied in neurotypical and language impaired populations across all age groups and it is well established that it reflects lexico-semantic processing (see Kutas & Federmeier, 2011 for a review). Despite the great amount of research, some controversies still exist around the functional interpretation of the N400. According to the *semantic integration* view, N400 amplitude indexes the difficulty with which a given word can be integrated into previous context, that is, how plausible it is (Brown and Hagoort, 1993, Coch et al., 2013, Hagoort et al., 2004, Van Berkum et al., 1999). In contrast, the *lexical activation/retrieval* account assumes that words are pre-activated by the prior context and that the N400 amplitude indicates the degree to which a certain word was expected or pre-activated (Lau et al., 2008, Brouwer et al., 2012, Van Berkum et al., 2009). More recently, a *hybrid* account has been proposed, suggesting that both activation and integration costs are reflected in the N400 (Baggio and Hagoort, 2011, Fritz and Baggio, 2020, Lau et al., 2016, Nieuwland et al., 2020). Accordingly, the N400 is argued to comprise non-compositional processes related to word predictability and pre-activation as well as compositional processes related to plausibility and integration into sentence context (Nieuwland et al., 2020). Of note, the present study does not aim to distinguish between these accounts. Instead, we assume the hybrid account as the best approximation and thus take the N400 to be modulated as a degree of both activation and integration.

While most previous studies have used whole sentence contexts to investigate the N400 and semantic composition effects, recent research has adapted a minimal composition paradigm (Fló et al., 2020, Fritz and Baggio, 2020, Lau et al., 2016, Neufeld et al., 2016). The seminal magnetoencephalography (MEG) study by Bemis & Pylkkänen (2011) presented participants with two-word phrases and compared brain activity when the stimuli could be composed (*red boat*) to when composition was not possible (*xkq boat*). Compositional phrases engaged the left anterior temporal lobe (ATL), the ventromedial prefrontal cortex (vmPFC) and sometimes the angular gyrus (AG) (Bemis and Pylkkänen, 2011, Bemis and Pylkkänen, 2013). These results have been replicated and extended to other modalities and languages (Blanco-Elorrieta et al., 2018, Pylkkänen et al., 2014, Westerlund et al., 2015). More recently, an electroencephalography (EEG) study observed a “combinatorial” N400, that is, a more negative wave for compositional phrases (*red boat*) versus non-compositional (*xkq boat/yerl boat*) (Neufeld et al., 2016). This effect was taken to reflect increased semantic combinatorial operations that are only possible in the real-word condition. However, a recent study aiming to replicate these results failed to find the same effect (Fló et al., 2020). Another study that aimed to distinguish the predictability (lexical access) and plausibility (integration) accounts in minimal combinatorial contexts found N400 effects for both unpredictable and implausible two-word phrases, with overall larger effects for unpredictable phrases (Lau et al., 2016). These results were taken to support the hybrid account of the N400.

Interestingly, another recent minimal combinatorial ERP study found a P600 effect for semantic composition instead of compositional activity in the N400 time-window (Fritz and Baggio, 2020). These results are in line with a general shift away from the idea that semantic processing is restricted to the N400 time-window and provide evidence for a semantic P600 effect (Aurnhammer et al., 2021, Brothers et al., 2020, Delogu et al., 2019, DeLong et al., 2014, Kuperberg et al., 2020). The P600 has initially been linked to syntactic processes such as re-analysis or repair of grammatically ill-formed sentences (Brown and Hagoort, 1993, Friederici et al., 1998, Hagoort, 2003, Osterhout and Holcomb, 1992) but more recently, some studies have attributed it to more controlled semantic processes such as semantic integration or repair (Brouwer et al., 2012, Kuperberg et al., 2020, Brouwer et al., 2017). Furthermore, the distinction into a frontal and a parietal late positivity has refined the understanding of the P600^2^ (Brothers et al., 2020, Kuperberg et al., 2020, Van Petten and Luka, 2012). While the frontal P600 has been shown to arise in response to unexpected but plausible sentence endings, the parietal P600 is maximal after highly implausible sentence continuations. However, the functional interpretation of the P600 in minimal combinatorial contexts remains less clear and further evidence is needed.

A related question that has not been addressed to date is whether the ERP changes between healthy participants and PWA found at the sentence level (Chang et al., 2016, Kawohl et al., 2010, Khachatryan et al., 2017, Sheppard et al., 2017, Swaab et al., 1997) are also present in minimal compositional paradigms. Although such differences are of no direct consequence for the clinical care of PWA, deficits in basic semantic composition may well contribute to the understanding of the overall comprehension deficit in aphasia. Several electrophysiological ‘biomarkers’ have been shown to differentiate between PWA and neurotypical controls and to correlate with aphasia severity. Despite the ongoing debate about the functional interpretation of the P600 (as discussed above), there is general agreement that both the N400 and P600 are strongly related to language processing and are therefore prime candidates in the diagnosis and monitoring of aphasia (Meechan et al., 2021). Specifically, the reduced and delayed N400 effect may serve as a potential diagnostic tool for aphasia. Notably, a detailed single case study in a participant with non-fluent aphasia showed correlations between the clinical improvement and the ‘normalization’ of different ERP components, including the N400 (Aerts et al., 2015). Thus, it seems justified to further explore ERP-signatures in aphasic speech production and comprehension, although this field is still in its infancy and needs to be improved in consistency. In particular, the current literature on the P600 profile in PWA is scarce. A better understanding of aberrant electrophysiological correlates of semantic composition in PWA may further elucidate the nature of the underlying deficit because ERPs might be more sensitive than behavioural data. Investigating changes in the temporal dynamics of ERP components across the time course of aphasia recovery after stroke could ultimately inform language rehabilitation research. Our minimal composition paradigm reduces the complexity of the linguistic material to two-word phrases and a simple two-choice judgment. This may allow for application even in more severely impaired PWA. Moreover, such minimal paradigms limit the effect of confounding factors during sentence processing such as attention, working memory and executive control (Maran et al., 2022).

In the present study, we employed a novel strategy to investigate the electrophysiological correlates of basic semantic composition by using both plausibility and lexicality manipulations. People with post-stroke aphasia and age-matched healthy controls were presented three types of adjective-noun phrases: *meaningful* (“anxious horse”), *anomalous* (“anxious wood”) and *pseudoword* phrases in which the noun is replaced by a pseudoword (“anxious terk”). Importantly, the adjective is matched across the three conditions, thus always triggering compositional processes while only the noun differs with respect to plausibility (*anomalous* versus *meaningful*) and lexicality (*pseudoword* versus *meaningful*). In a previous lesion study with the same paradigm, we observed that PWA with lesions in left anterior inferior frontal gyrus (aIFG) had difficulties in correctly judging the plausibility of anomalous phrases, while lesions to left ATL impacted their response times on those phrases specifically (Graessner et al., 2021).

The main goal of the present study was to characterize semantic composition difficulties in PWA at the temporal level. Assuming a hybrid account of the N400, we expected that neurotypical controls would show an equally strong N400 effect towards anomalous and pseudoword phrases as compared to meaningful phrases, indicating a difficulty to integrate the unexpected word/pseudoword into the phrase. Given the deficits in judging anomalous phrases, (Graessner et al., 2021) we expected that PWA would show a reduced and/or delayed N400 effect towards anomalous phrases. At the same time, as their pseudoword judgment was relatively unimpaired, we expected the N400 effect towards pseudowords to be comparable to that of the control group. We did not have a clear hypothesis about a potential P600 effect, as the literature on minimal phrases and the P600 is still scarce. However, based on sentence processing studies, a tentative hypothesis was to expect a P600 following the N400 towards anomalous phrases at least in the control group. We note that our study was exploratory regarding potential differences in the P600 in PWA.

## 2 Materials and methods

### 2.1 Participants

The PWA-group consisted of 20 right-handed (self-reported, prior to stroke), native German speaking participants with an acquired chronic left-hemispheric lesion (female = 9; mean age = 58 years; age range = 48–67 years, mean months since onset = 78, range = 20–170). One additional patient was tested but not included in the analyses due to responses given prior to the response-window. An age-matched group of 20 right-handed, native German speaking participants served as controls (female = 11; mean age = 58 years; age-range = 47–70 years). In the control group, two additional participants were tested but were excluded later due to excessive artifacts. The participants from the age-matched control group were additionally administered the Mini-Mental Status Test (MMST) (Kessler et al., 1990) to screen for mild cognitive impairment (MCI). All participants had a score > 26/30 making MCI improbable and ensuring that they are neurologically typical.

**Fig. 1:**
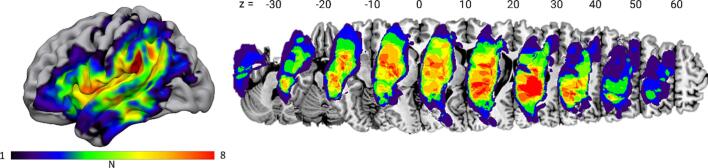
Lesion overlap map. The colour scale ranges from 1 to 8 lesions. Coordinates refer to z-values in MNI space.

In the PWA-group, all lesions were of vascular origin including ischemic and hemorrhagic infarction. The aphasia profiles included a large range of aphasia types and severities as classified by the Aachener Aphasia Test (AAT) (Huber et al., 1983) and the speech and language therapists’ (SLT) judgment. Table 1 provides an overview. In case of disagreement between the AAT and SLT-judgment, the latter is listed. The classification was as follows: 5 Broca’s, 3 Amnestic, 1 Wernicke’s, 3 non-classifiable aphasias and 8 PWA with residual aphasia. Note that due to the probabilistic classification of syndromes in the AAT, ‘non-classifiable’ means that none of the 4 standard syndromes (Broca’s, Wernicke’s, Amnestic/ Global) exceeded 70 % probability. ‘*[syndrome]*/ NCL’ indicates that probabilities for a standard syndrome were close to 70 %. Since aphasia severity is judged by the Token Test and the profile-height in the different tests participants with a low error rate in the Token Test are judged as ‘no or residual aphasia’ although they may show clear deficits in subtests. In these participants the major deficit is provided (e.g., phon^deficit^ means clear deficits in the subtests regarding phonological competence). More demographic information on the PWA-group can be found in Table 1 and a lesion overlap map is shown in Fig. 1. All participants gave their written informed consent and were financially compensated for their effort. The study protocol conformed to the principles of the Declaration of Helsinki and was approved by the local ethics committee at the University of Leipzig (reference 155/17-ek and 251/18-ek). All participants were recruited from the database of the Max Planck Institute for Human Cognitive and Brain Sciences and the Clinic for Cognitive Neurology, University Hospital Leipzig. Exclusion criteria were severe overall cognitive impairment and pre-morbid left-handedness. All participants from the PWA group had participated in a previous study with the identical paradigm (Graessner et al., 2021) at least 6 months prior to the recruitment for this study.

**Table 1:** Patient demographics.

**sex**		**age /yrs**	**etiol**	**hem**	**LesVol /cm^3^**	**MSO**	**type**	**severity**	**TT/ %rank**	**LexDec / %**	**Synon / %**	**NVST /%**
**f**		49	Isch	L	7.7	134	rep^deficits^	resid	99	95	92.5	96
**f**		67	ICH	L	9.9	62	sem^deficit^	resid	95	98.8	90.0	96
**f**		61	ICH	L	10.7	41	sem^deficit^	resid	99	97.5	90.0	83
**m**		49	ICH	L	15.9	68	AMN	mild/mod	79	86.3	82.5	100
**f**		64	Isch	L	24.4	53	sem^deficit^	resid	99	96.3	92.5	96
**f**		56	SAH/Isch	L	28.3	99	NCL	mild	99	97.5	95.0	97
**m**		52	SAH/Isch	L	32.4	56	sem^deficit^	resid	97	98.8	90.0	100
**m**		59	Isch	L > R	33.6	41	NCL	mod	67	87.5	85.0	100
**m**		61	Isch	L	37.3	119	AMN/NCL	mild/mod	95	96.3	90.0	97
**m**		59	ICH	L	37.9	90	BRO/NCL	mild	76	87.5	85.0	96
**f**		67	Isch	L	38.0	152	phon^deficits^	resid	99	96.3	92.5	92
**m**		57	Isch/TBI	L	41.2	70	syn^deficits^	resid	97	92.5	75.0	83
**m**		48	Isch	L	46.5	69	NCL	mod	58	91.3	80.0	97
**f**		57	Isch	L	53.6	80	BRO/NCL	mild	93	95.0	75.0	92
**f**		54	Isch	L	60.8	20	phon^deficits^	resid	97	93.8	85.0	94
**m**		53	Isch	L	104.0	94	AMN	mild	99	88.8	55.0	100
**m**		55	Isch	L	130.1	34	BRO	mild	91	82.5	90.0	*NA*
**m**		65	Isch	L	144.9	33	WER	mod	47	88.8	77.5	92
**m**		65	Isch	L	164.9	170	BRO	mod	74	86.3	92.5	100
**f**		57	Isch	L	426.3	85	BRO	sev	51	92.5	87.5	*NA*
**9f**	**mean**	**57.8**			**72.4**	**78.5**			**85.6**	**92.4**	**85.1**	**95.1**
**11m**	*SD*	*5.79*			*92.61*	*39.57*			*17.02*	*4.70*	*9.06*	*5.04*

**etiol** = Etiology, **hem** = lesioned hemisphere, **MSO** = months since onset, **TT** = percentage rank based on age-corrected errors in the Tokentest, **LesVol** = Lesionvolume in cm^3^, **Lex Dec / Synon** = lexical decision (80 items) and synonym judgement (40 items) of the LeMo diagnostics given in % correct, **NVST** = % correct in the nonverbal semantic test, **Isch** = ischemia, **SAH** = subarachnoidal hemorrhage, **ICH** = intracerebral hemorrhage, **TBI** = traumatic brain injury, **BRO** = Broca’s, **WER** = Wernicke’s, **AMN** = amnestic, **NCL** = non classifiable aphasia. **rep^deficts^**/ **sem^deficts^** / **syn^deficts^** / **phon^deficts^** = for residual aphasias: deficits most prominent for repetition / lexico-semantics / syntax/ phonology; **mild**/ **mod** / **sev** / **resid** = mild / moderate / severe / residual severity of aphasia, NA = data not available.

### 2.2 Experimental paradigm

All participants performed one experimental session of about 90 min including EEG preparation time. After fitting the EEG cap, participants performed a 10 min “baseline” task followed by the main experimental task. The baseline task was designed to elicit a robust N400 effect for pseudowords versus words (Aerts et al., 2015, Bentin, 1987, Friedrich et al., 2006, Kutas and Federmeier, 2000). This task consisted of 50 single words and 50 single pseudowords that were not included in the main experimental task. Participants were instructed to press a button upon hearing a direct repetition of items to ensure constant attention. Ten percent of the items (i.e., 5 pseudo- / 5 words) served as catch trials and were excluded from further analyses. The task was practiced in a training block prior to the start of the experiment. As the baseline task did not involve combinatorial semantics, results are not reported here.

**Fig. 2:**
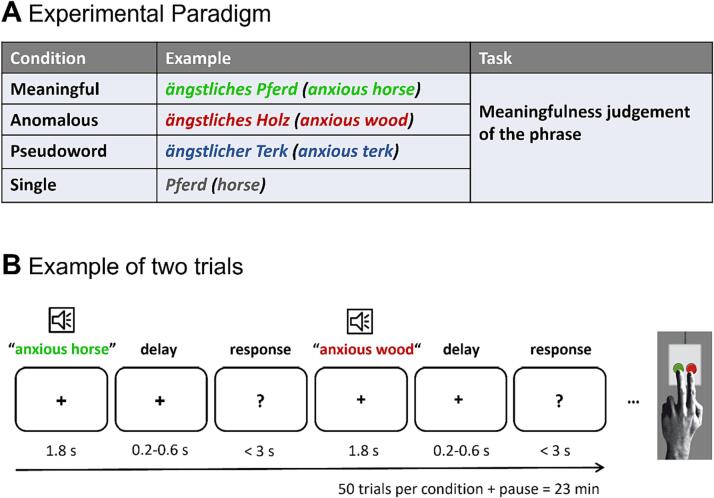
Experimental Design. **A** Experimental conditions and task description used in the study. **B** Example of two trials. The delay period was jittered from 200 ms up to 600 ms with a mean duration of 400 ms.

The main experimental task was identical to the one used in our previous studies (Graessner et al., 2021, Graessner et al., 2021). Stimuli were presented auditorily via loudspeakers, and participants were asked to judge the meaningfulness of each phrase by forced binary choice button press (meaningful/not meaningful) with the index or middle finger of their left hand. Stimuli consisted of spoken word pairs that were either meaningful (“anxious horse”), anomalous (“anxious wood”) or had the noun replaced by a pseudoword (“anxious terk”). Additional single word stimuli (“horse”) served as low-level baseline and were included to balance responses, so that 50 % of the stimuli required a ‘meaningful’ (meaningful and single words) and 50 % a ‘not meaningful’ (anomalous and pseudowords) response. After each stimulus, participants had to withhold their response for a variable delay of 200–600 ms to prevent motor confounds within the time window of interest (van Vliet et al., 2014). They were asked to respond as quickly and accurately as possible upon seeing a question mark appear on screen. Responses prior to the appearance of the question mark were not included in the analysis of EEG data, but accuracy of those trials was analyzed. Timeout for responses was after 3 s. Speaker volume was adjusted to a comfortable level and a practice block checked for comprehension of task requirements. The experimental session consisted of five blocks with all conditions appearing 10 times in each block. Stimulus order was pseudo-randomized across participants. Blocks were separated by rest periods of at least 20 s and participants could continue the experiment via button press. Stimuli were presented using the software Presentation (Neurobehavioral Systems, Inc., Albany, CA, USA). Fig. 2 illustrates the paradigm and timing.

### 2.3 Stimuli

The material included three experimental and one baseline condition: (i) The *meaningful* condition (e.g., “anxious horse”) allows for successful semantic integration; (ii) The *anomalous* condition comprises two meaningful words which cannot be semantically integrated based on world knowledge (e.g., “anxious wood”), since the adjective violates the selectional restriction criteria of the noun. This condition triggers the attempt of meaning composition which should fail in case the lexico-semantic system is intact; (iii) For the pseudoword condition, the noun was replaced by a pseudoword as generated by the software Wuggy (Keuleers and Brysbaert, 2010) (e.g., “anxious terk”) which prevents both lexical retrieval and integration. Note that the syntactic structure is kept identical for these three conditions. This avoids confounds by a different number of words or differences in syntactic complexity while selectively varying the amount of semantic information. Lastly, a *single word* condition (iv) was included as a low-level baseline and to match the number of ‘meaningful’ and ‘not meaningful’ judgments. The final set of stimuli consisted of 50 phrases per condition, matched for word frequency, orthographic neighborhood, length, gender, and concreteness (for more details and a list of all stimuli, see Supplementary Materials).

### 2.4 Data acquisition and preprocessing

Data were collected in a dimly lit, electrically shielded, soundproof chamber at the Max Planck Institute for Human Cognitive and Brain Sciences, Leipzig. EEG was recorded from 63 active Ag/AgCl electrodes attached according to the 10–20 system in an elastic cap connected to a Refa8 amplifier. The left mastoid served as reference electrode during recording. The ground electrode was placed on the sternum. Offline re-referencing was performed with the average signal of the left and right mastoids. Horizontal and vertical eye movements (EOG) were monitored with four electrodes placed above and below the left eye and on the outer sides of both eyes. Impedances were always kept below 10 k Ω in the control group. In the PWA-group a few participants exceeded this threshold and impedances below 20 k Ω were accepted. EEG signals were digitized with a sampling rate of 500 Hz. The EEG data were preprocessed and analyzed using the Matlab FieldTrip toolbox (Oostenveld et al., 2011).

The data were filtered offline with a Kaiser-windowed finite-impulse response high-pass filter with half-amplitude cutoff of 0.3 Hz and a low-pass filter with a half-amplitude cutoff of 30 Hz. We subsequently segmented and time-locked the data to the onset of the noun, with a 200 ms pre-stimulus period and 1300 ms post-noun-onset period. Artifact rejection was performed semi-automatically. Segments of the signal exceeding a z-value of 10 were highlighted automatically and were then screened manually to reject artifacts (e.g., muscle artifacts). Ocular artifacts were corrected by applying an independent component analysis (ICA), that decomposed the data from all channels into 62 components and subsequent manual rejection of components corresponding to blinks and saccades. Trials in which participants responded incorrectly did not enter the analysis, because the origin of the error can be at different levels (attentional, lexical and compositional). On average, 13.8 % (SD 9.48 %) of trials were rejected in the PWA-group and 7.9 % (SD 5 %) in the control group. As the rejection rate differed significantly between groups (*p* < 0.05), we used single trial-based data in our statistical models, following the approach by Frömer et al. (2018) who provided one of the first processing pipelines for single trial-based analyses of EEG data with linear mixed models. According to best-practice guidelines for N400 studies (Šoškić et al., 2022), the most common minimum number of trials per condition is 30. However, due to the difficulty in recruiting participants with aphasia during the Covid-19 pandemic, we also included five participants with less than 30 trials in one condition (n = 2 with 29 trials, n = 2 with 27 trials, n = 1 with 20 trials). Mean number of trials per condition was 43 in the PWA-group and 46 in the control group. Baseline correction on the remaining trials was applied using the average signal in the 200 ms interval prior to the onset of the noun. Since the single word condition served as baseline and to balance responses, we did not include it in the ERP analyses. Note that in this condition no phrase is presented, rendering an analysis of semantic composition meaningless. For completeness and as a check for whether participants correctly understood the task, the behavioral data are reported also for the single word condition.

### 2.5 Data analysis

#### 2.5.1 Behavioral data

For statistical analysis of the behavioral data, we used generalized linear mixed effects models (GLMMs), as implemented in the lme4 package (Bates et al., 2015) in R (version 4.1.1; R Core Team, 2021), fitting a binomial model for accuracy. As we asked participants to delay their response until they saw the response cue on screen, response times (RTs) might be influenced by inhibitory control mechanisms. We nevertheless analyzed RTs to investigate potential condition and group effects and modeled RTs assuming a Gamma distribution with an identity link function. As suggested by Lo & Andrews (2015), GLMMs can account for the distribution of RT data (right-skewed with a long tail) without the need to transform the raw data. A gamma distribution best describes the shape of RT data, and the identity link function simply describes that no transformation was done (every element is mapped to itself). In both models, we included by-participant intercepts to account for overall inter-individual differences. Additionally, we modeled by-item intercepts. Condition and group served as fixed effects, and we added an interaction term. We used sum-coding for the predictors and report main effects and interactions. To follow-up condition-by-group interactions, we used the *emmeans* package (Lenth et al., 2018) and conditioned the pairwise comparisons on group and condition, respectively.

#### 2.5.2 EEG data

For the statistical analyses of the EEG data, we first used non-parametric cluster-based permutation tests (CBPTs) (Maris and Oostenveld, 2007) to identify within-group N400 and P600 effects. Based on previous studies on the N400 effect in the aging and lesioned brain (Khachatryan et al., 2017, Lucas et al., 2019, Poulisse et al., 2020, Tiedt et al., 2020) and after visual inspection of our waveforms, the N400 amplitude was determined in the window 350–550 ms. For the P600 effect, we analyzed the time-window from 600 to 1000 ms. For each time window, we conducted CBPTs for the condition pairs *anomalous* versus *meaningful* and *pseudoword* versus *meaningful* using the dependent sample T-statistic. The cluster-level statistic was calculated as the maximum of the cluster-level summed T-values of each cluster. Clusters were required to have at least two neighboring channels and two neighboring time points. As we expected a negative cluster for the N400 time window and a positive cluster for the P600 time window for the contrasts described above, one-tailed tests were conducted and the critical alpha level for the Monte Carlo significance probability was set to 0.05. The permutation distribution was based on 1000 permutations.

To investigate differences in the amplitude of the effects between groups, we conducted single trial based linear mixed models (LMMs) for average amplitude over the two pre-defined time windows. For the N400 effect, we thus extracted single-trial amplitudes between 350 and 550 ms from the pre-defined electrodes positions F3, Fz, F4, C3, Cz, C4, P3, Pz, P4 (based on the set of electrodes most often reported in N400 studies; see Šoškić et al. (2022). For the P600 time window, as we did not have clear expectations whether to expect a frontal or a parietal effect, we considered a frontal and a parietal set of electrodes separately, over the time-window from 600 to 1000 ms (frontal: FPz, FP1, FP2, AFz, AF3, AF4, AF7, AF8; parietal: Pz, P1, P2, P3, P4, POz, PO3, PO4, Oz, O1, O2; according to Wang et al. (2021). In each model, we added condition and group as fixed effects and participant and item as random intercepts. We did not include random slopes in our models as this led to convergence errors. To follow-up condition-by-group interactions and to investigate whether the respective effect was larger in one group than in the other, we created the contrasts of interest (*anomalous* versus *meaningful* and *pseudoword* versus *meaningful* for condition and *PWA* versus *controls* for group) using the *hypr* package (Rabe et al., 2020, Schad et al., November 2018). Statistical significance of the effects was evaluated using the *lmerTest* package (Kuznetsova et al., 2017).

The N400 in PWA has been shown to be not only decreased in amplitude but also delayed (Meechan et al., 2021). Therefore, we analyzed latency differences using the fractional area latency method (Kiesel et al., 2008, Liesefeld, 2018) as recommended by Sassenhagen & Draschkow (2019). This method has been found to be robust against high-frequency noise, in contrast to the often used peak latency (Liesefeld, 2018). To this end, the N400 latency was defined as the time point when the component reached 50 % of its area under the curve. We set the time-window to 300–600 ms to account for individual differences in latency. Because components that occur rather late might be far off from the pre-stimulus baseline, parts of the area might not actually belong to the component. We therefore adjusted the baseline to 30 % of the component amplitude to avoid contamination by low-amplitude activity and adjacent components, as recommended by Liesefeld (2018). This resulted in one value per participant per condition (averaged across the 9 pre-selected electrodes) and we passed the values as the dependent variable to an ANOVA with group and condition as fixed effects. Note that here we are not interested in the latency of the difference wave between conditions (N400 *effect*) but in the latency of the components for each condition (N400 *amplitude*).

Finally, we conducted exploratory correlation analyses to investigate the relationship between N400 and P600 effects within each group. We therefore ran Pearson’s correlations on the N400 and P600 effects for *anomalous* versus *meaningful* and *pseudoword* versus *meaningful* respectively. To this end, we took the single subject average difference over 350–550 ms from the 9 electrodes selected for each N400 effect separately and correlated this with a) the single subject average difference over 600–1000 ms from the frontal electrodes for the frontal P600 and b) the single subject average difference over 600–1000 ms from the parietal electrodes for the parietal P600. Additionally, we correlated the individual token test scores of the PWA with their ERP effects. However, as no correlations showed significant effects, we present these results in the Supplementary Materials.

## 3 Results

### 3.1 Behavioral results

**Fig. 3:**
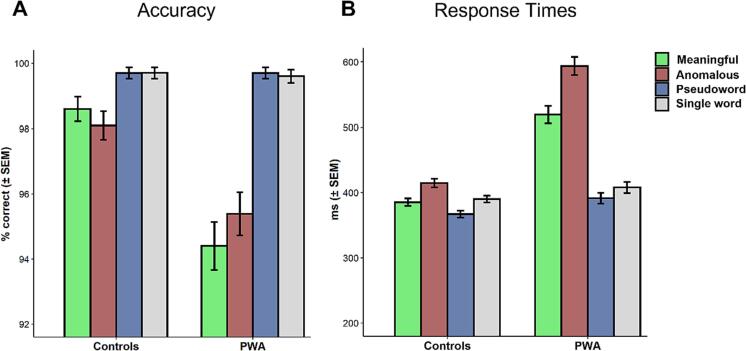
Accuracy (A) and response times (B) for both groups.

Overall, mean accuracy was high in both groups (controls = 99 %, PWA = 97 %), indicating that task demands were low for all participants (Fig. 3A). The GLMM yielded a significant main effect for group indicating the expected lower performance in PWA (F(1) = 8.427, *p* < 0.005) and a main effect of condition (F(3) = 19.187, *p* < 0.001), with meaningful and anomalous phrases showing lower accuracy as compared to pseudoword phrases and single words in both groups. There was no significant interaction between group and condition (F(3) = 1.422, *p* = 0.197).

Response times were overall faster in the control- compared to the PWA-group (Fig. 3B). The GLMM revealed strong main effects for group (F(1) = 8.97, *p* < 0.001) and condition (F(3) = 34.791, *p* < 0.001), as well as an interaction (F(3) = 47.45, *p* < 0.001). Follow-up comparisons between groups confirmed that PWA responded slower in all conditions (all *p* < 0.001) except for the single word condition. Within-group comparisons showed that in the control group, responses to anomalous phrases were significantly slower than to meaningful (*p* < 0.005) and pseudoword phrases (*p* < 0.001). Responses to pseudoword phrases were overall fastest and showed a trend towards being significantly faster than to single words (*p* = 0.07). The pattern was qualitatively similar in the PWA group, with overall larger differences between conditions. Statistical comparisons were significant for all pairwise comparisons expect for the contrast of pseudowords versus single words (*p* = 0.39, all other *p* < 0.001, see Table 2 for full model output).

**Table 2:** Model output for follow-up analyses on response times.

**Within-group effects: Response Times**				
**group = Controls**	***estimate***	***SE***	***z***	***p***
**M vs. A**	−31.12	8.38	−3.712	**< 0.005**
**M vs. P**	8.69	7.96	1.091	0.695
**M vs. S**	−13.41	10.0	−1.341	0.537
**A vs. P**	39.80	6.40	6.216	**< 0.001**
**A vs. S**	17.71	8.93	1.984	0.194
**P vs. S**	–22.09	9.10	−2.429	0.07

**group = PWA**	***estimate***	***SE***	***z***	***p***

**M vs. A**	−67.15	8.40	−7.996	**< 0.001**
**M vs. P**	92.93	7.49	12.407	**< 0.001**
**M vs. S**	77.91	10.06	7.744	**< 0.001**
**A vs. P**	160.08	7.88	20.321	**< 0.001**
**A vs. S**	145.06	9.48	15.306	**< 0.001**
**P vs. S**	−15.02	9.55	−1.573	0.394

**Between-group effects: Response Times**				

**Controls-PWA**	***estimate***	***SE***	***z***	***p***

**M**	−115.7	14.8	−7.828	**< 0.001**
**A**	−151.7	16.4	−9.243	**< 0.001**
**P**	−31.4	14.7	−2.144	**< 0.03**
**S**	−24.4	15.1	−1.611	0.107

A = anomalous, M = meaningful, P = pseudowords, S = single words.

### 3.2 ERP results

**Fig. 4:**
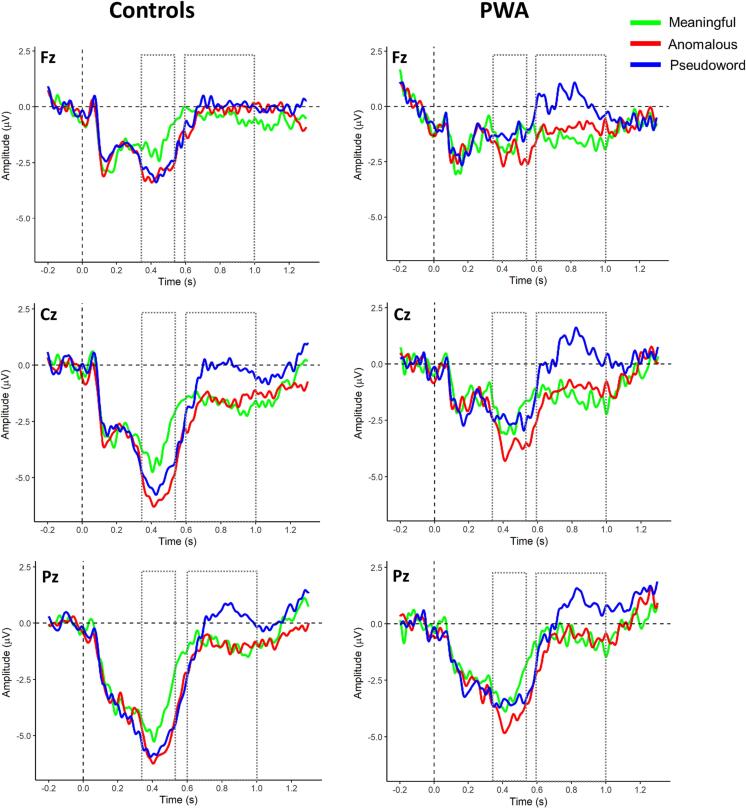
Grand average waveforms at three representative electrodes for the control group (left) and the PWA-group (right). The dashed vertical line marks noun onset. Dotted rectangles mark the time-windows for the N400 (350–550 ms) and P600 (600–1000 ms) analyses.

Fig. 4 presents the grand-average ERP waveforms for meaningful, anomalous and pseudoword phrases at selected electrodes for both groups. Visual inspection revealed the typical N1-P2 pattern in response to auditory stimuli, which is noisier in the PWA-group. In both groups, a centro-parietal negativity peaking around 400 ms (N400) can be observed in all conditions. Results for the late positive component (P600) are more heterogeneous. Descriptively, pseudoword phrases show the most pronounced P600 effect. For the other conditions, the component is less clearly discernible. Notably, the pattern differs between PWA and control group. This is evident most distinctly for the response to pseudoword phrases over frontal electrodes, which shows a pronounced P600 in the PWA- but not in the control group.

In the following, we present the results of the N400 and P600 *effects*, indicating the difference between ERPs in response to the anomalous and pseudoword condition when compared to the meaningful condition. Please note that larger efforts for semantic composition should result in a more negative N400-effect and a more positive P600-effect.

#### 3.2.1 N400 effect within groups (CBPT)

**Fig. 5:**
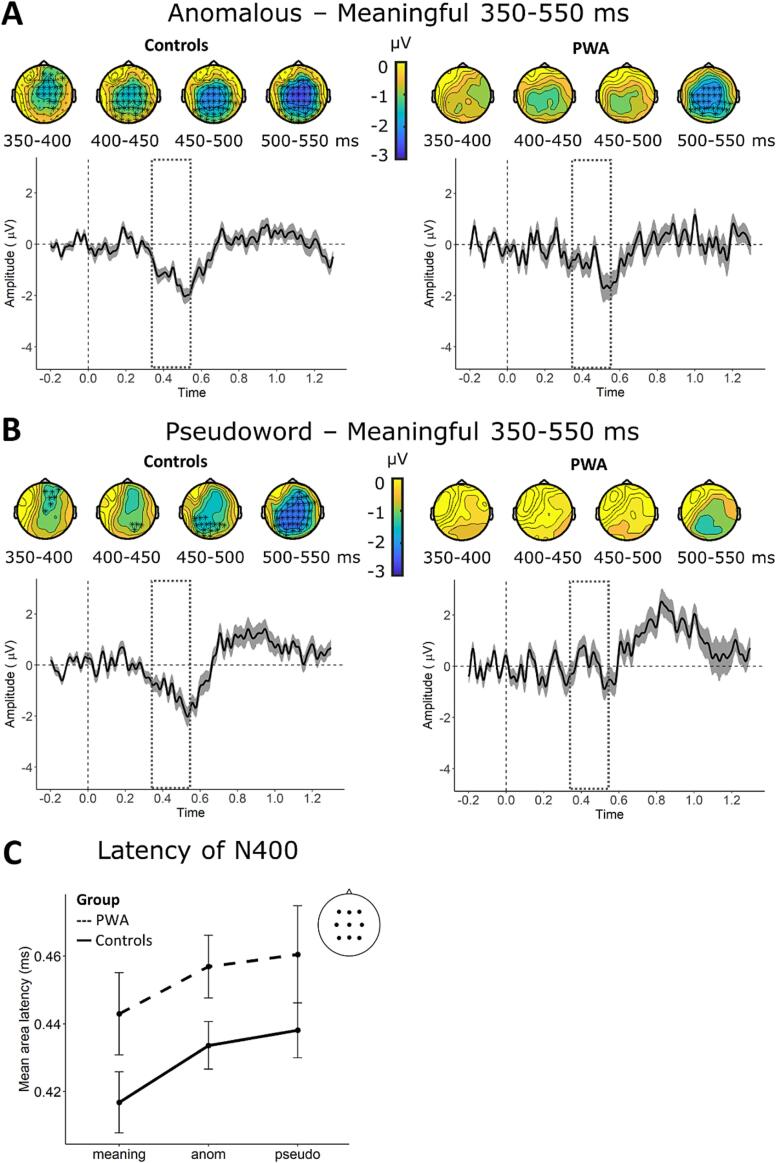
Scalp topographies for 50 ms time bins for anomalous versus meaningful phrases (**A top**) and pseudoword versus meaningful (**B top**) with electrode clusters based on which the null hypothesis in the CBPT was rejected highlighted with asterisks. Below the topographies, ERP difference waveforms averaged across 9 electrodes (Fz, F3, F4, Cz, C3, C4, Pz, P3, P4) with shaded area reflecting 95 % confidence intervals are depicted; the dashed line indicates the time-window for which single trial LMMs of amplitude differences were calculated. (**C**) Mean fractional area latency of the N400 per condition and group obtained at 9 fronto-centro-parietal electrodes. Error bars indicate standard error of the mean (SEM).

Fig. 5 shows the difference waves between the response to anomalous (A) and pseudoword phrases (B) after subtraction of the response to meaningful phrases. The N400 effect for anomalous phrases is clearly observed for both groups, while the effect pseudoword phrases is only seen for the control group. The CBPTs in the latency range from 350 to 550 ms confirm these results: In the control group, we found significant effects for anomalous (*p* < 0.001) and for pseudoword phrases (*p* < 0.005), while the PWA group only showed an effect for anomalous phrases (*p* < 0.05). Fig. 5 additionally depicts the scalp-distributions across 50 ms time bins within the N400 time-window, highlighting electrode clusters based on which the null hypothesis was rejected, indicating a significant N400 effect.

#### 3.2.2 N400 effect between groups (LMM)

The linear mixed-effects model revealed a main effect for condition (F(2) = 6.551, *p* < 0.005), a marginally significant main effect for group (F(1) = 3.941, *p* = 0.054) and an interaction of group and condition (F(2) = 4.078, *p* < 0.05). The contrast coding showed no group difference for the anomalous effect (estimate = 0.515, SE = 0.452 t = 1.139, *p* = 0.255). Conversely, groups differed for the pseudoword effect (estimate = 1.288, SE = 0.454, t = 2.836, *p* < 0.005), in that controls showed a stronger N400 effect than PWA (for full model output see Table 4). A summary of mean amplitudes over the selected electrodes from 350 to 550 ms for condition and group can be found in Table 3.

**Table 3:** Mean amplitudes in µV across selected electrodes for the respective time-windows.

Condition	N400 amplitude (SD)		Frontal P600 amplitude (SD)		Parietal P600 amplitude (SD)	
	**Controls**	**PWA**	**Controls**	**PWA**	**Controls**	**PWA**
Meaningful	−2.78 (5.71)	−2.35 (8.25)	−1.06 (6.59)	−2.47 (9.31)	−0.51 (4.73)	−0.37 (7.3)
Anomalous	−4.21 (5.37)	−3.33 (8.09)	−0.91 (6.23)	−1.99 (9.59)	−0.52 (4.52)	−0.62 (6.88)
Pseudowords	−3.96 (5.57)	−2.3 (8.38)	−1.26 (5.68)	−1.05 (8.8)	0.26 (4.65)	0.44 (6.73)

**Table 4:** ERP mixed-model output.

**N400 time window**	***estimate***	***SE***	***z***	***p***
(Intercept)	−3.1246	0.2849	−10.967	**< 0.0001**
A vs. M	−1.1679	0.3230	−3.615	**< 0.0005**
P vs. M	−0.5374	0.3243	−1.657	0.0995
group: PWA vs. Control	1.0669	0.5375	1.985	0.0543
A vs. M: PWA vs. Control	0.5150	0.4521	1.139	0.2546
P vs. M: PWA vs. Control	1.2882	0.4543	2.836	**< 0.005**

**Late frontal positivity**	***estimate***	***SE***	***z***	***p***

(Intercept)	−1.4358	0.3810	−3.769	**< 0.001**
A vs. M	0.3386	0.3354	1.009	0.3143
P vs. M	0.6159	0.3367	1.829	0.0693
group: PWA vs. Control	−0.7728	0.7396	−1.045	0.3026
A vs. M: PWA vs. Control	0.4313	0.5002	0.862	0.3885
P vs. M: PWA vs. Control	1.7228	0.5026	3.428	**< 0.001**

**Late parietal positivity**	***estimate***	***SE***	***z***	***p***

(Intercept)	−0.23550	0.24078	−0.978	0.3329
A vs. M	−0.06900	0.27910	−0.247	0.8051
P vs. M	0.85477	0.28022	3.050	**< 0.005**
group: PWA vs. Control	0.03911	0.45094	0.087	0.9313
A vs. M: PWA vs. Control	−0.18024	0.37668	−0.478	0.6323
P vs. M: PWA vs. Control	0.10122	0.37850	0.267	0.7891

A = anomalous, M = meaningful, P = pseudowords, S = single words.

#### 3.2.3 N400 Latency differences between groups

The analysis of latency differences between groups revealed a significant main effect of group (F(1) = 8.17, *p* < 0.01) but neither the main effect for condition nor the interaction for group × condition were significant. As illustrated in Fig. 5C, the N400 is delayed in PWA for all conditions.

#### 3.2.4 P600 effect within groups (CBPT)

**Fig. 6:**
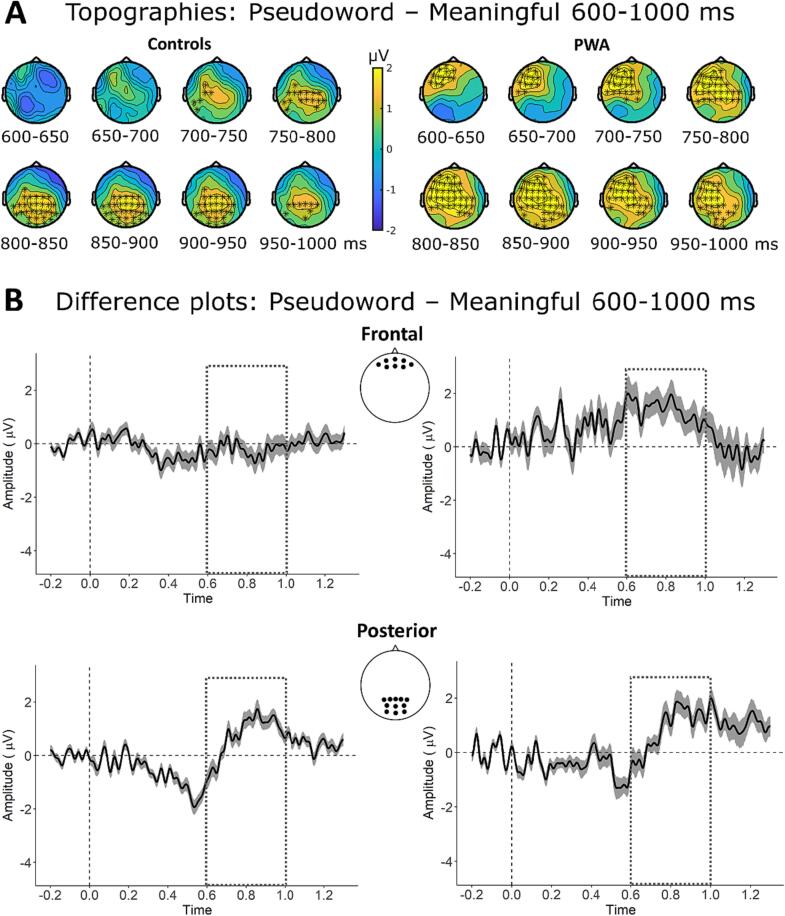
Scalp topographies in 50 ms time bins from 600 to 1000 ms for pseudoword – meaningful phrases with electrode clusters based on which the null hypothesis in CBPT was rejected highlighted with asterisks. **B** ERP difference waveforms averaged across frontal (Fpz, Fp1, Fp2, AF7, AF8, Afz, AF3, AF4) and parietal electrodes (Pz, P1, P2, P3, P4, POz, PO3, PO4, Oz, O1, O2) with shaded area reflecting 95 % confidence intervals; the dashed line indicates the time-window for which single trial LMMs of amplitude differences were calculated.

Visual inspection of the grand-averaged ERP waveforms for the P600 time-window (Fig. 4) revealed a positivity for pseudoword phrases over parietal electrodes in both groups and additionally over frontal electrodes in the PWA group. The CBPTs confirmed these results and revealed significant differences between pseudoword and meaningful phrases in both groups with a more frontal distribution in the PWA group (both *p* < 0.05). For the contrast anomalous versus meaningful phrases, there were no positive clusters in either group (both *p* > 0.14). The scalp distributions shown in Fig. 6 illustrate the topographical differences of the effects in the two groups and highlight the electrodes contributing to the largest cluster in the CBPT.

#### 3.2.5 P600 amplitude effect between groups (LMM)

The LMM showed an interaction of group and condition for frontal electrodes (F(2) = 6.38, *p* < 0.005). The contrast coding revealed no significant group difference for the anomalous effect (estimate = 0.431, *SE* = 0.50, *t* = 0.862, *p* = 0.388), but a significant difference for pseudoword effect (estimate = 1.723, *SE* = 0.503 *t* = 3.428, *p* < 0.001) with PWA showing a stronger frontal positivity than controls. At parietal electrodes, only a main effect of condition was significant, indicating that the responses to pseudowords were more positive than that to meaningful phrases in both groups (estimate = 0.854, *SE* = 0.280, *t* = 3.050, *p* < 0.005; see Table 4 for full model output). A summary of mean amplitudes over frontal and parietal electrodes from 600 to 1000 ms for condition and group can be found in Table 3.

#### 3.2.6 Correlation of N400 and P600 amplitude

**Fig. 7:**
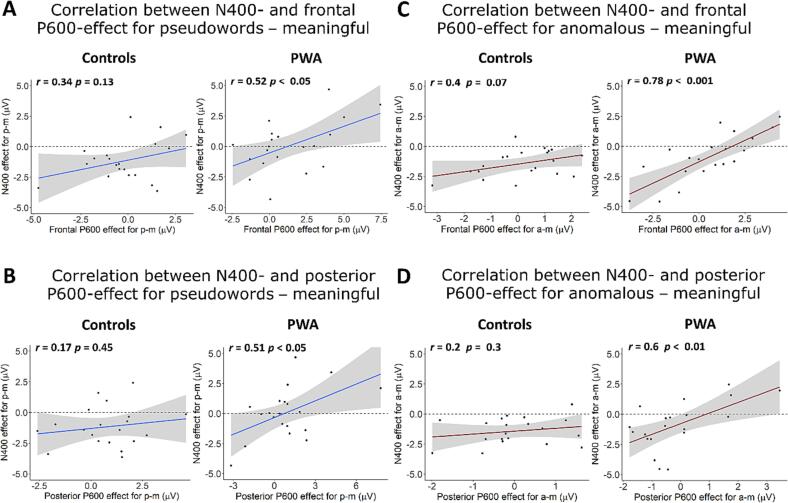
Correlation between N400 and P600 effects. **A** and **B** show frontal and parietal P600 effect correlations with the N400 effect for pseudoword – meaningful phrases. **C** and **D** show frontal and parietal P600 effect correlations with the N400 effect for anomalous phrases. Error bands indicate SEM. The horizontal dashed line serves as a visual aid for whether an N400 effect was present in an individual (points below the line) or not present (points on or above the line). a = anomalous, m = meaningful, p = pseudoword.

Finally, we performed an exploratory analysis on the relationship between the amplitudes of the N400 and P600 effects. We used Pearson’s correlations across participants’ means to test whether N400 effects correlated with frontal and/or parietal P600 effects. This was done separately for both groups. Given that the N400 effect is negative and the P600 effect is positive, a positive correlation would mean that the smaller the N400 effect, the larger the P600 effect. In the control group, neither the correlation for the pseudoword N400 effect with the frontal nor the parietal P600 effect was significant (both *p* > 0.1; see Fig. 7 A and B). Conversely, in the PWA group, both frontal and parietal P600 effects correlated positively with the pseudoword N400 effect (both *r*> = 0.51, *p* < 0.05). The correlation for the anomalous N400-effect showed a trend towards significance for frontal electrodes in the control group (r = 0.4, p = 0.07) and was strong for both the frontal and parietal P600 effect in the PWA group (both *r*> = 0.6, *p* < 0.01; see Fig. 7 C and D). Taken together, a smaller N400 effect for both anomalous and pseudoword phrases predicted larger late P600 effects in PWA.

## 4 Discussion

In this study, we investigated differences in the electrophysiological responses during a basic semantic composition task in people with aphasia (PWA) and age-matched controls. We focused on the N400 and P600 effects, both indexing lexical-semantic processing. These effects were assessed as the difference between the ERP-responses towards anomalous (“anxious wood”) or pseudoword phrases (“anxious terk”) and the ERP-response to semantically plausible meaningful phrases (“anxious horse”). To our knowledge, this is the first study that compares PWA and age-matched healthy controls with a paradigm that manipulates both lexicality as well as plausibility in a minimal phrase with a constant syntactic structure (adjective-noun).

We found an N400 effect for anomalous phrases with a typical centro-parietal distribution in both groups. The N400 component was delayed for all conditions in PWA when compared to age-matched controls. Crucially, despite accurate and fast responses towards pseudoword phrases, PWA did not show an N400 effect, which was prominent in the control group. In the P600 time-window, both groups exhibited an effect with larger positivity for pseudoword phrases over parietal electrodes. No such positivity was observed for anomalous phrases, which speaks for a later processing step in the P600 time window that is specific for pseudowords. Unlike controls, PWA showed an additional P600 effect over frontal electrodes towards pseudoword phrases. Finally, only in the PWA group, we found a correlation of N400 and P600 effects (frontal and parietal), indicating that patients with no or even reversed N400 effects towards pseudoword and anomalous phrases showed a stronger late positivity. Together, the results illustrate that basic semantic composition is altered in people with an acquired brain lesion. Behaviorally, PWA showed less correct and slower responses for the anomalous and the meaningful conditions, while electrophysiologically, additional changes during semantic composition could be demonstrated also pertaining to the processing of pseudoword phrases.

### 4.1 Automatic semantic mismatch detection is unimpaired in PWA

Despite the significant behavioral difference between PWA and the control group on anomalous phrases, we did not observe an amplitude difference for the N400 effect of anomalous phrases between groups. This suggests that the automatic semantic mismatch detection is intact (although slightly delayed), while later explicit plausibility judgement is specifically impaired in this group of patients. This finding is in line with previous priming studies showing intact automatic semantic access but impaired explicit semantic judgements in PWA (Hillert, 2004, Milberg and Blumstein, 1981, Ostrin and Tyler, 1993, Salles et al., 2012). We note that the N400 effect towards anomalous phrases is unlikely to arise merely due to lexical prediction effects but may additionally reflect fast and automatic integration attempts. Given previous evidence that the N400 reduction in PWA depends on aphasia severity (Chang et al., 2016, Kawohl et al., 2010, Swaab et al., 1997, Hagoort et al., 1996), the largely preserved N400 in the present cohort is plausible due to the relatively good recovery of our cohort, which overall showed very mild to moderate aphasia at the time of inclusion. Notably, however, the plausibility judgment on anomalous and meaningful phrases was challenging for the PWA-group as evidenced by lower accuracy and speed of their judgement. The impaired performance on anomalous and meaningful phrases in PWA suggests that their impairment affects semantic composition rather than lexicality judgment. Although we assessed ERPs in response to the second constituent (i.e., the noun), the very good performance regarding the pseudoword condition suggests that PWA were able to access the meaning of both constituents of the phrase but had difficulties to correctly judge on a composite meaning. The preserved N400 effect may reflect the fact that the frequency of anomalous phrases like “anxious wood” is extremely low compared to phrases such as “anxious horse”. The judgement on pseudoword phrases on the contrary was largely unimpaired. This underlines the relatively spared lexical abilities in the PWA cohort, by which the lack of meaning for pseudowords eases the judgment on the respective phrase. This will be discussed in the following paragraph. Please note that our study used a delayed response cue, therefore, an ERP effect potentially correlating to the late plausibility judgment was not accessible.

### 4.2 No N400 effect towards pseudoword phrases in PWA

While we expected that pseudoword processing in PWA should be unimpaired, we in fact observed no N400 effect towards pseudowords in PWA. This is surprising given the preserved behavioral performance on pseudoword phrases. A possible explanation for the lack of an N400 effect is that due to rather well-preserved lexical abilities, PWA recognize the pseudoword as meaningless automatically, and do not spend cognitive resources on trying to integrate the pseudoword into a phrase. Their correct and fast judgement on pseudoword phrases may rely on a superficial lexical-semantic processing at this time-point, while neurotypical participants can use their unimpaired resources to perform early lexical and compositional processing. Bearing in mind that PWA perform just as accurately on judging the plausibility of pseudoword phrases as the control group, a compensatory mechanism may correct for the initial “skip” of the pseudoword in the N400 time window to indicate that this word cannot be retrieved and integrated (see discussion below). A potential alternative explanation under the lexical activation/retrieval account could be that PWA show reduced semantic activation for pseudowords in the N400 time-window coupled with a strong task-modulated P600. Note, however, that as our paradigm was not designed to distinguish between the different N400 accounts, both alternative explanations for the missing N400 effect in PWA are subject to further testing and neither can be conclusively confirmed or rejected at this stage.

Additionally, it should be noted that previous studies reported spatio-temporal component overlap between the N400 and the P600, resulting in a “cancelling out” between the two components (Brouwer and Crocker, 2017, Luck, 2014). We are confident that our effects cannot be explained in terms of a cancelling out effect since we separated the time windows for the analyses by at least 50 ms and used different electrode clusters. Consequently, we minimized potential component overlap to the best possible degree.

Previous ERP studies on aphasic language processing mainly investigated responses to semantic anomalies and found reduced N400 effects in PWA (Chang et al., 2016, Kawohl et al., 2010, Khachatryan et al., 2017, Sheppard et al., 2017, Swaab et al., 1997). The few studies that compared real-word with pseudoword responses proposed an “aphasia recovery potential” that is specific to words (Pulvermüller et al., 2004, Pulvermüller et al., 2005). These studies showed that after therapy, an early ERP response changed for words but not for pseudowords (Pulvermüller et al., 2005). Conversely, a more recent study with a passive lexical detection experiment showed that PWA had a comparable N400 effect towards pseudowords as age-matched controls (Aerts et al., 2015). However, besides the implicit nature of the stimulus presentation, this study is not directly comparable to our study, as only acute aphasic patients were included. It is thus up to future research to confirm our finding that pseudoword phrases do not elicit an N400 in PWA, despite fast and accurate behavioral responses.

### 4.3 Support for the hybrid N400 account

Although our design did not aim to differentiate between the different accounts of the N400, we believe that our data support the *hybrid* account (Baggio and Hagoort, 2011, Fritz and Baggio, 2020, Lau et al., 2016, Nieuwland et al., 2020). The control group showed an equally strong negativity towards anomalous and pseudoword phrases, indicating that the N400 response does not differentiate between the two types of stimuli. However, if the N400 were just a response at the lexical level and merely reflected retrieval difficulties or prediction error, in line with the classical graded N400 effect (Boudewyn et al., 2015, Wlotko and Federmeier, 2013, Federmeier and Kutas, 1999), we should observe the largest N400 towards pseudoword phrases with a gradient of a smaller N400 effect to anomalous phrases. Pseudowords cannot be predicted, while anomalous words might profit from some pre-activation due to semantic feature overlap with meaningful word continuations (i.e., anomalous words are also concrete words). The absence of a graded N400 pattern in the control group speaks for both lexical retrieval and integration processes to cause the N400 effect. This means that an early automatic attempt for semantic integration is reflected in this time window, which fails for both the integration of the anomalous word and the pseudoword.

### 4.4 No P600 effect towards anomalous phrases in both groups

Prior research on semantic anomalies at the sentence level has shown that two late positivities following the N400 are indicative of semantic re-processing or even repair (DeLong et al., 2014, Kuperberg et al., 2020, Van Petten and Luka, 2012, Quante et al., 2018). The late frontal P600 is elicited by unexpected but plausible sentence continuations such as “After proposing, he put the ring on her *dresser*”, and is thought to reflect the detection of a lexical prediction violation, the integration of new and previously unexpected information into the current representation of meaning, or both (Brothers et al., 2020, Federmeier et al., 2007). In contrast, the late parietal P600 is usually elicited by highly implausible sentence continuations (e.g. “Every morning for breakfast the eggs would *eat*…”) and is linked to conflict detection and prolonged attempts to repair the input (Kuperberg, 2007). Several studies have further refined the functional interpretation of these positivities by showing that they are both strongly dependent on context (Brothers et al., 2020, Kuperberg et al., 2020). However, a recent high-powered study has called the clear distinction between the two positivities into question (Stone et al., 2023). Here, the authors aimed to replicate the effect that the frontal positivity is affected by constraint at unexpected but plausible words, while the N400 and the parietal positivity are not. Instead, they found an effect for the parietal positivity, suggesting that previous findings might have been an artifact of smaller sample size. Crucially, in all experiments, real-word anomalies in multi-sentence contexts were used but pseudowords were not included in the stimulus material. Our minimal phrases have inherently very sparse context and predictions can only be based on a single adjective. According to the *hierarchical generative framework* (Brothers et al., 2020, Kuperberg et al., 2020), we may assume that our participants would only build a minimal model that includes just enough levels of representation to perform the task. This minimal model includes semantic and syntactic features but, critically, does not contain a higher-level situation model which is necessary to elicit re-analysis processes as is assumed to be reflected by the P600 (Brothers et al., 2020). Overall, it seems that in this minimal context, the semantic evaluation for anomalous phrases is completed within the N400 time-window.

### 4.5 The P600 effect is specific for pseudowords

Our finding of a parietal P600 towards pseudowords in both the control group and PWA could not be predicted by the hierarchical generative framework, which is only based on real words. Previous studies that used pseudoword sentences focused mainly on the syntactic P600 effect and often removed all semantic content from the stimuli by presenting so-called *jabberwocky* sentences (Ericsson et al., 2008, Hahne and Jescheniak, 2001).

Probably closest to our design is the recent minimal combinatorial literature, but a critical difference lies in the order of words: As in the original *red boat* paradigm (Bemis and Pylkkänen, 2011), the following adaptations created the non-combinatorial phrase by substituting the first word with either a nonword (*xqg*) or a pseudoword (*yerl*) (Fló et al., 2020, Fritz and Baggio, 2020, Neufeld et al., 2016, Markiewicz et al., 2021). The few existing studies come to different conclusions regarding putative ERP correlates of semantic composition: While Neufeld et al. (2016) found a stronger N400 towards compositional (*blue car*) than non-compositional phrases (*rnsh/yerl car*) in the composition task, Flo et al. (2020) claimed that this effect was due to the blocked task design inducing expectancy processes. Finally, Fritz & Baggio (2020) found a (parietal) P600 in response to semantic conditions as compared to nonword- or pseudoword phrases, which was taken to reflect phrasal composition. Crucially, they also observed an early difference between the two conditions at around 200 ms, which could be interpreted as an attentional modulation.

None of these minimal combinatorial studies thus found a classical P600 effect towards semantic anomalies indicating reanalysis processes, probably stemming from the different word order and possibly due to different attentional processes depending on the lexical status of the first word. In our paradigm, however, participants did not know before noun onset whether they would hear a real word or a pseudoword and compositional processes were thus always triggered by the adjective. Consequently, we did not observe early attention-guided differences. Rather, differences between conditions started in the N400 time-window and lasted up to 1 s after noun-onset. We therefore suggest that our parietal P600 effect reflects the failed attempt for reanalysis or repair.

The additional frontal positivity in PWA might be due to an anterior shift in ERP components, as observed in previous aphasia studies, indicating that PWA rely more strongly on executive functions (Chang et al., 2016, Angrilli et al., 2003, Chiappetta et al., 2022, Wilson et al., 2012). It is also likely that the frontal P600 is dependent on the N400 effect and arises only in case of a missing N400 effect (see discussion below). The more widespread P600 effect over frontal and parietal electrodes in PWA is also in line with a study by Khachatryan et al. (2017) who observed similar N400 effects but increased P600 effects in PWA as compared to age-matched controls. They suggest that PWA rely more on semantic processing in the later time-window requiring conscious efforts rather than fully automatic processes.

### 4.6 The P600 effect as potential compensatory mechanism

Our correlation results demonstrate that those people with aphasia who did not exhibit an N400 effect towards pseudoword or anomalous phrases show a stronger frontal and parietal P600. No significant relationship was found in the control group. PWA showed an N400 effect for anomalous phrases, yielding a significant effect at the group level. Conversely, for pseudoword phrases, there was no group-level N400 effect but instead a frontal and a parietal P600 effect. The strong correlation of N400 and P600 effects in PWA might indicate a compensatory mechanism: When automatic early processing is impaired, PWA make use of a more elaborate mechanism trying to reanalyze the meaning of the phrase. This compensatory mechanism seems to be independent of the violation type (anomalous or pseudoword phrase) and might help PWA to successfully identify that composition of these phrases is impossible. Notably, it has previously been suggested that the P600 is modulated by task relevance (Contier et al., 2021, Schacht et al., 2014, Vissers et al., 2007). It would therefore be interesting to test in future studies whether we observe the same positivities in the absence of a plausibility judgment task.

Finally, some previous imaging studies reported significant correlations between aphasia severity and neuroimaging readouts (DeMarco et al., 2021, Kawano et al., 2021, Li et al., 2017). In contrast to these studies, we did not find any significant correlations between individual ERP effects and aphasia severity, as measured by the token test (see Supplementary Fig. 1).

## 5 Future directions and limitations

A potential clinical application for the here presented stimulus material could be to support diagnostic specificity. The material goes beyond simple lexical retrieval but stays at a complexity level that is manageable even with severe aphasia. The degree to which basic semantic composition is impaired could thus indicate the necessity for further diagnostic testing.

As a limitation of our study, we note that our sample size is relatively small (although similar to previous studies on PWA). We had to stop data collection due to the Covid-19 pandemic. In addition to overall greater statistical power, an advantage with larger sample sizes could be to divide the patient group into anterior and posterior lesions and explore differences between these subgroups. This would be particularly interesting as a previous lesion-symptom mapping study from our group (Graessner et al., 2021) found differential effects for frontal and anterior temporal lesions. Nevertheless, both studies suggest convergent evidence of a semantic composition deficit from different methodologies.

Additionally, it should be noted that the cohorts of the present study include older adults only, while most of the previous studies on the N400 and P600 effects have been conducted in young adults. The present results are therefore not generalizable to the typical N400 population and future studies should include a young control group for comparison.

## 6 Conclusion

Overall, our study provides new evidence for a unique difference in pseudoword processing in people with aphasia relative to healthy controls. The N400 amplitude effect for anomalous phrases did not differ between groups, indicating spared automatic semantic composition. Crucially, PWA did not show an N400 effect for pseudowords phrases, but instead a strong late frontal and parietal P600. While healthy controls showed both an N400 and a parietal P600, the frontal P600 unique to PWA might be part of a successful compensation strategy. Whether the positivity is purely task-dependent remains to be clarified in future research.

## CRediT authorship contribution statement

**Astrid Graessner:** Conceptualization, Investigation, Data curation, Formal analysis, Visualization, Writing – original draft, Writing – review & editing. **Caroline Duchow:** Investigation, Formal analysis, Writing – review & editing. **Emiliano Zaccarella:** Conceptualization, Writing – review & editing. **Angela D. Friederici:** Conceptualization, Writing – review & editing. **Hellmuth Obrig:** Conceptualization, Writing – review & editing. **Gesa Hartwigsen:** Conceptualization, Funding acquisition, Supervision, Writing – review & editing.

## Declaration of Competing Interest

The authors declare that they have no known competing financial interests or personal relationships that could have appeared to influence the work reported in this paper.

## Data Availability

Data will be made available on request.

## Footnotes

2 Throughout this paper, we will refer to the late positivities as the P600 for easier readability.
