# Supplementary material for: Electrophysiological correlates of basic semantic composition in people with aphasia
Source: Neuroimage Clin. 2023 Sep 23;40:103516. doi: 10.1016/j.nicl.2023.103516 (PMC10540050; doi:10.1016/j.nicl.2023.103516)
Supplement: Supplementary data 1 [file mmc1.docx]

**Supplementary Materials and Methods**

**Supplementary Table 1.** Psycholinguistic variables for the two real-word conditions.

|  | Anomalous | Meaningful | *p* |
| --- | --- | --- | --- |
| **Adjectives** | | | |
| Frequency | 2.04 (0.96) | 2.12 (0.96) | 0.753 |
| OLD-20 | 2.00 (0.46) | 1.91 (0.42) | 0.434 |
| **Nouns** | | | |
| Frequency | 2.56 (0.57) | 2.58 (0.55) | 0.810 |
| OLD-20 | 1.66 (0.33) | 1.68 (0.32) | 0.604 |
| Concreteness | 4.87 (0.12) | 4.86 (0.15) | 0.585 |
| **Pairs** |  |  |  |
| Meaningfulness rating | 1.51 (0.29) | 5.53 (0.34) | **< 0.0001** |

Frequency and OLD-20 (orthographic neighborhood) measures were taken from the SUBTLEX-DE database and frequency is given as log-transformed per 1 million words. Concreteness was determined using concreteness ratings for 40.000 English words. Ratings were obtained from 20 participants who did not take part in the experiment. Numbers in brackets represent standard deviation.

**Supplementary Table 2.** Stimulus list for anomalous, meaningful and pseudoword phrases

| **Condition** | **Stimulus (english translation)** | **Mean Plausibility Rating** |
| --- | --- | --- |
| **A** | nervoeser Salat (anxious salad) | 1.05 |
| **A** | aengstliches Holz (anxious wood) | 1.1 |
| **A** | braver Lappen (obedient cloth) | 1.1 |
| **A** | felsiges Schaf (rocky sheep) | 1.1 |
| **A** | fluessiges Auto (liquid car) | 1.1 |
| **A** | gestimmtes Schwein (tuned pig) | 1.1 |
| **A** | ruhiger Krug (quiet jug) | 1.1 |
| **A** | stumpfer Fisch (blunt fish) | 1.1 |
| **A** | zahmes Brett (tame board) | 1.1 |
| **A** | koestliches Schiff (delicious ship) | 1.2 |
| **A** | morsches Kamel (rotten camel) | 1.2 |
| **A** | toter Korb (dead basket) | 1.2 |
| **A** | blinder Finger (blind finger) | 1.25 |
| **A** | frisches Dreieck (fresh triangle) | 1.25 |
| **A** | hoelzerner Schaum (wooden foam) | 1.25 |
| **A** | lautes Auge (loud eye) | 1.25 |
| **A** | modischer Spinat (fashionable spinach) | 1.25 |
| **A** | mutiger Knochen (brave bone) | 1.25 |
| **A** | poroeser Schwan (porose swan) | 1.25 |
| **A** | sportliches Glas (sporty glass) | 1.25 |
| **A** | stolzes Kabel (proud wire) | 1.25 |
| **A** | pikanter Teppich (spicy carpet) | 1.3 |
| **A** | behaarter Ofen (hairy oven) | 1.35 |
| **A** | defekter Loewe (defective lion) | 1.35 |
| **A** | hungriges Klavier (hungry piano) | 1.35 |
| **A** | luftiger Teller (airy plate) | 1.35 |
| **A** | ovales Pferd (oval horse) | 1.35 |
| **A** | eckiges Pferd (rectangular horse) | 1.4 |
| **A** | lockerer Pfau (loose peacock) | 1.45 |
| **A** | defekter Fuchs (defective fox) | 1.5 |
| **A** | einsamer Motor (lonely engine) | 1.5 |
| **A** | leerer Guertel (empty belt) | 1.5 |
| **A** | senkrechter Helm (vertical helmet) | 1.5 |
| **A** | wilder Koffer (wild suitcase) | 1.5 |
| **A** | zackiger Schlauch (pointed tube) | 1.5 |
| **A** | biegsamer Mond (flexible moon) | 1.55 |
| **A** | faules Kleid (lazy dress) | 1.55 |
| **A** | junger Kittel (young smock) | 1.55 |
| **A** | bequemer Zahn (comfortable tooth) | 1.6 |
| **A** | wilder Stuhl (wild chair) | 1.6 |
| **A** | dummer Schal (stupid scarf) | 1.65 |
| **A** | stabiler Wolf (stable wolf) | 1.65 |
| **A** | weiblicher Tisch (female table) | 1.7 |
| **A** | freches Paket (naughty package) | 1.8 |
| **A** | wachsames Plakat (vigilant poster) | 1.85 |
| **A** | unscharfes Hemd (blurry shirt) | 1.9 |
| **A** | steinerner Brief (stone letter) | 1.95 |
| **A** | stoerrischer Berg (stubborn mountain) | 2.05 |
| **A** | eiserner Pilz (iron mushroom) | 2.2 |
| **A** | rostiger Vogel (rusty bird) | 2.25 |
| **M** | ruhiger Motor (quiet engine) | 4.65 |
| **M** | einsamer Vogel (lonely bird) | 4.75 |
| **M** | felsiger Berg (rocky mountain) | 4.85 |
| **M** | ovaler Teller (oval plate) | 4.85 |
| **M** | spitzes Dreieck (pointy triangle) | 4.95 |
| **M** | braves Schaf (obedient sheep) | 5.1 |
| **M** | senkrechter Pfosten (vertical pole) | 5.1 |
| **M** | stolzer Pfau (proud peacock) | 5.2 |
| **M** | poroeser Knochen (porose bone) | 5.25 |
| **M** | steinernes Kreuz (stone cross) | 5.25 |
| **M** | toter Wolf (dead wolf) | 5.25 |
| **M** | defekter Schlauch (defective tube) | 5.3 |
| **M** | hohler Zahn (hollow tooth) | 5.3 |
| **M** | hoelzernes Schiff (wooden ship) | 5.35 |
| **M** | zahmer Loewe (tame lion) | 5.35 |
| **M** | aengstliches Pferd (anxious horse) | 5.4 |
| **M** | eiserner Ofen (iron oven) | 5.45 |
| **M** | frecher Affe (naughty monkey) | 5.5 |
| **M** | morscher Baum (rotten tree) | 5.5 |
| **M** | sturer Esel (stubborn donkey) | 5.5 |
| **M** | handliches Paket (compact package) | 5.55 |
| **M** | hungriger Fuchs (hungry fox) | 5.55 |
| **M** | koestlicher Salat (delicious salad) | 5.55 |
| **M** | leeres Plakat (blank poster) | 5.55 |
| **M** | luftiges Kleid (airy dress) | 5.55 |
| **M** | weiblicher Schwan (female swan) | 5.55 |
| **M** | biegsames Kabel (flexible wire) | 5.6 |
| **M** | dummes Schaf (stupid sheep) | 5.6 |
| **M** | lautes Auto (loud car) | 5.6 |
| **M** | offener Beutel (open bag) | 5.6 |
| **M** | duenner Pinsel (thin brush) | 5.65 |
| **M** | krummer Finger (crooked finger) | 5.65 |
| **M** | blindes Auge (blind eye) | 5.7 |
| **M** | giftiger Pilz (poiseneous mushroom) | 5.7 |
| **M** | schmaler Guertel (narrow belt) | 5.7 |
| **M** | rostiges Schwert (rusty sword) | 5.8 |
| **M** | sauberes Glas (clean glass) | 5.8 |
| **M** | bequemes Hemd (comfortable shirt) | 5.85 |
| **M** | junger Hirsch (young deer) | 5.85 |
| **M** | leerer Koffer (empty suitcase) | 5.85 |
| **M** | runder Mond (round moon) | 5.85 |
| **M** | stabiles Regal (stable shelf) | 5.85 |
| **M** | blutiges Knie (bloody knee) | 5.9 |
| **M** | eckiger Tisch (rectangular table) | 5.9 |
| **M** | gestimmtes Klavier (tuned piano) | 5.9 |
| **M** | kaputtes Fahrrad (broken bicycle) | 5.9 |
| **M** | reifer Kaese (mature cheese) | 5.9 |
| **M** | modischer Schal (fashionable scarf) | 5.95 |
| **M** | bequemer Stuhl (comfortable chair) | 6 |
| **M** | unscharfes Foto (blurry photo) | 6 |
| **P** | blindes Urto (blind Urto) |  |
| **P** | blutiger Terk (bloody Terk) |  |
| **P** | braver Dinder (obedient Dinder) |  |
| **P** | duennes Ulge (thin Ulge) |  |
| **P** | eckiges Pravier (rectangular Pravier) |  |
| **P** | einsames Braft (lonely Braft) |  |
| **P** | eisernes Nafor (iron Nafor) |  |
| **P** | fauler Krauf (lazy Krauf) |  |
| **P** | felsiger Orfe (rocky Orfe) |  |
| **P** | fluessiger Samtel (liquid Samtel) |  |
| **P** | frecher Bive (naughty Bive) |  |
| **P** | frischer Drosten (fresh Drosten) |  |
| **P** | gestimmter Pern (tuned Pern) |  |
| **P** | giftiges Pleient (poiseneous Pleient) |  |
| **P** | handlicher Odel (compact Odel) |  |
| **P** | hoelzerner Forb (wooden Forb) |  |
| **P** | hohles Manel (hollow Manel) |  |
| **P** | hungriger Kon (hungry Kon) |  |
| **P** | koestlicher Taun (delicious Taun) |  |
| **P** | krummes Safel (crooked Safel) |  |
| **P** | luftiger Herk (airy Herk) |  |
| **P** | modisches Fenn (fashionable Fenn) |  |
| **P** | morscher Pirtel (rotten Pirtel) |  |
| **P** | mutiges Jaehn (brave Jaehn) |  |
| **P** | nervoeses Pleut (anxious Pleut) |  |
| **P** | offener Masat (open Masat( |  |
| **P** | ovaler Struk (oval Struk) |  |
| **P** | pikantes Sprenn (spicy Sprenn) |  |
| **P** | poroeses Kriel (porose Kriel) |  |
| **P** | reifes Segil (mature Segil) |  |
| **P** | rostiger Nader (rusty Nader) |  |
| **P** | ruhiges Schreif (quiet Schreif) |  |
| **P** | rundes Baset (round Baset) |  |
| **P** | sauberer Noller (clean Noller) |  |
| **P** | schmaler Godel (narrow Godel) |  |
| **P** | senkrechter Juersch (vertical Juersch) |  |
| **P** | spitzer Gartel (pointy Gartel) |  |
| **P** | sportlicher Kolk (sporty Kolk) |  |
| **P** | stabiler Eken (stable Eken) |  |
| **P** | steinerner Drau (stone Drau) |  |
| **P** | stoerrischer Pflaut (stubborn Pflaut) |  |
| **P** | stolzer Fippen (proud Fippen) |  |
| **P** | stumpfer Grinat (blunt Grinat) |  |
| **P** | sturer Lotis (stubborn Lotis) |  |
| **P** | totes Schmick (dead Schmick) |  |
| **P** | unscharfer Glant (blurry Glant) |  |
| **P** | wachsamer Liffer (vigilant Liffer) |  |
| **P** | weiblicher Tehrt (female Tehrt) |  |
| **P** | zackiger Phles (pointed Phles) |  |
| **P** | zahmer Naeppich (tame Naeppich) |  |

A = Anomalous, M = Meaningful, P = Pseudoword. Ratings were obtained from 20 participants who did not take part in the experiment.

**English translation of the written task instructions**

„Welcome!

You will now hear several word pairs and your task is to decide whether a pair makes sense. It is not important how often you have actually seen the objects, but how plausible the combination seems to you.

Example: sharp knife = meaningful;

wooden foam = not meaningful.

Some word pairs contain pseudo-words. These words have no meaning and therefore do not make sense.

Example: green terk = not meaningful.

Some phrases contain single words. These are meaningful.

Knife = meaningful.

You will see a cross in the middle of the screen. Please focus on this cross at all times during the experiment.

Please only respond when a question mark appears. Once the question mark appears, respond as quickly and correctly as possible.

Now let's start with a brief practice block!“

**Supplementary Results**

There were no significant correlations between aphasia severity (as measured by the token test score) and N400 or P600 effects (see Supplementary Figure 1).


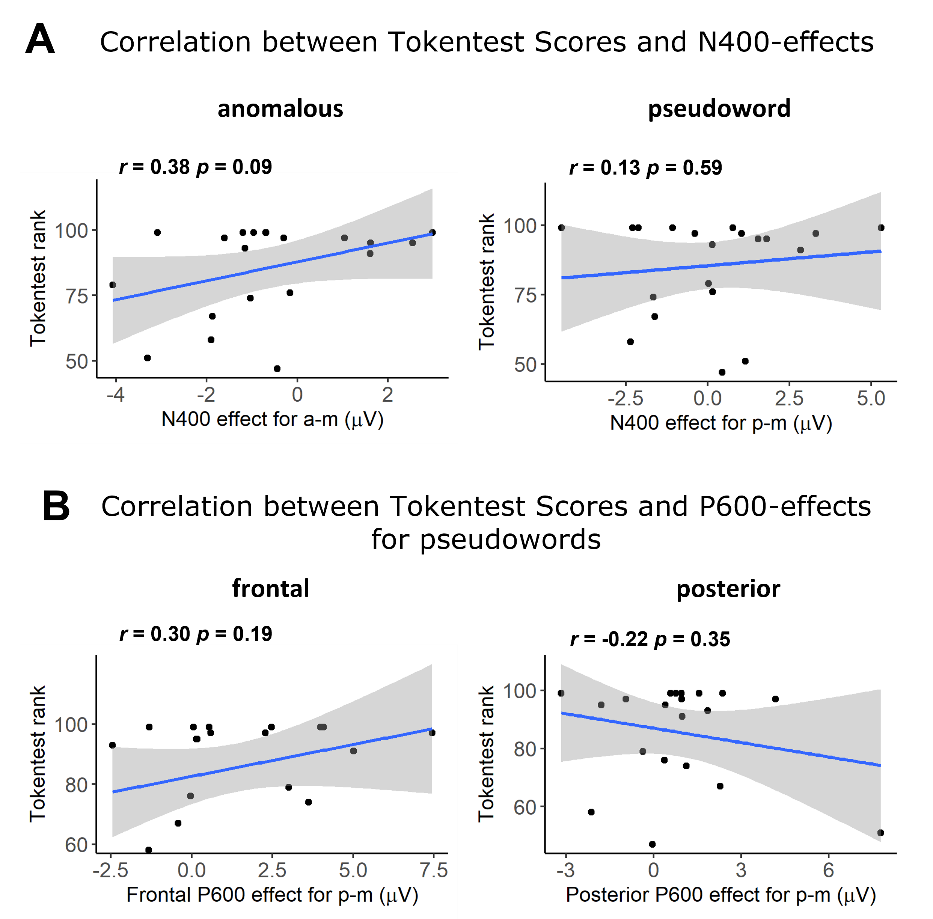


**Supplementary Figure 1.** Results of the exploratory correlation analyses between aphasia severity (as measured by the Token Test of the Aachen Aphasia Test) and ERP amplitudes.
